# Preliminary effectiveness of musical messaging to improve child eye health service uptake in Zanzibar: a pilot randomised trial

**DOI:** 10.1136/bmjopen-2025-107348

**Published:** 2025-09-23

**Authors:** Fatma Omar, Omar Juma Othman, Ai Chee Yong, Dina Belluigi, Christine Graham, Ronnie Graham, Eden Mashayo, Ving Fai Chan

**Affiliations:** 1 Ministry of Health, Zanzibar, United Republic of Tanzania; 2 Planning, Policy and Research, Zanzibar Ministry of Health, Zanzibar, United Republic of Tanzania; 3 Centre for Public Health, Queen’s University Belfast, Belfast, UK; 4 School of Social Sciences, Education and Social Work, Queen’s University Belfast, Belfast, Northern Ireland, UK; 5 Independent Researcher, Edinburgh, UK; 6 Vision Care Foundation, Dar-es-Salaam, United Republic of Tanzania

**Keywords:** Health Education, Community child health, PUBLIC HEALTH

## Abstract

**Objective:**

To assess the preliminary effectiveness and cost-effectiveness of a culturally tailored, music-based broadcast intervention delivered through schools and community radio to improve referral adherence among schoolchildren to inform the need for a definitive trial.

**Design:**

Pilot randomised interventional study.

**Setting:**

18 schools across Unguja and Pemba islands, Zanzibar.

**Participants:**

Schoolchildren (6–18 years old) who failed vision screening and were referred for care recruited from January to February 2024. The registered sample size reflects the full cohort, including children and adults. This manuscript reports on the child cohort only, as per the predefined analysis plan.

**Intervention:**

Group 1 received 3 months of school-based broadcasts of culturally tailored 3–6 min songs (played three times daily on 2 days per week), followed by 3 months of community radio broadcasts of additional songs (3–6 min, aired three times daily); Group 2 received the community broadcasts during the same period as Group 1.

**Primary and secondary outcomes:**

The primary outcome was change in referral adherence assessed at two time points: 3 months after school broadcast and 3 months after community broadcast, expressed in difference-in-difference estimates and effect sizes. Secondary outcomes included reporting of adverse events and contamination, and cost-effectiveness calculated as cost per child reached and cost per referred child accessed care in study groups and combined intervention.

**Results:**

374 children were referred to eye care services, including 246 in Group 1 and 128 in Group 2. Referral adherence was 69.8% in Group 1 and 42.9% in Group 2 (p=0.0006). The school broadcast phase yielded an effect size of 0.26 and a cost of US$4.65 per referred child accessing services. The community broadcast produced an effect size of 0.21, with a cost of US$0.29 per person reached. The combined intervention reached individuals at a cost of US$0.37 per person. No adverse event and contamination was reported.

**Conclusion:**

A combined school and community broadcast intervention improved referral adherence in this pilot trial, with evidence of cost-effectiveness. These findings support the conduct of a fully powered definitive trial.

**Trial registration number:**

NCT06469697.

**STRENGTHS AND LIMITATIONS OF THIS STUDY**

- The intervention was cocreated with community members, enhancing cultural relevance and feasibility.
- Contamination between groups was minimal, supported by follow-up checks with participants and implementers.
- Referral adherence was tracked through school records, clinical registers and coupons, though self-report may have introduced bias.
- Blinding of participants and outcome assessors was not possible, raising the risk of performance bias.
- Unequal school sizes, greater rural representation in one arm and delayed intervention rollout in Group 2 may have affected comparability and recall.

## Introduction

 Vision impairment affects approximately 19 million children globally, with uncorrected refractive error accounting for a substantial proportion of avoidable vision loss.^1^ School-based vision screening programmes have been widely implemented to identify children with vision impairments early; however, low adherence to follow-up referrals remains a persistent challenge. Studies across sub-Saharan Africa (SSA) report poor uptake of referral services following screenings, with adherence rates between 30% and 45% in settings such as Nigeria,^2^ Kenya^3^ and South Africa.^4^ Common barriers include parental misconceptions about children’s vision needs, concerns about treatment costs, logistical difficulties and sociocultural beliefs surrounding eye health.^5^

In Zanzibar, despite efforts to strengthen school eye health services through vertical models (stand-alone school eye health programmes) and integrated models (delivered alongside school feeding), substantial gaps persist. Chan *et al* demonstrated that even with an integrated delivery model, referral uptake was suboptimal, with many children identified through screening failing to complete follow-up care.^6^ A subsequent cost-effectiveness analysis similarly found that while integration into school health systems reduced costs compared with vertical models, ensuring treatment completion remained a challenge.^7^ Further qualitative research underscored the role of contextual factors in shaping referral adherence, such as competing school health priorities, parental awareness and health system bottlenecks.^8^ These findings highlight that, in Zanzibar as elsewhere in SSA, screening alone is insufficient: additional interventions are needed to bridge the gap between identification and treatment, ensuring children receive the care required to prevent avoidable vision loss.

Traditional approaches to eye health education in Zanzibar, such as posters and pamphlets, have had limited impact on promoting follow-up after school vision screenings.^9^ These methods often rely on passive information delivery and assume relatively high literacy and sustained engagement. In low-resource settings where literacy levels vary and printed materials are not widely accessible, posters and pamphlets may not capture attention effectively or motivate behaviour change. Moreover, cultural acceptability is a concern; posters were sometimes removed,^9^ suggesting they were perceived as inappropriate within community spaces. Unlike static health promotion methods, broadcast media potentially offered a dynamic and culturally resonant alternative.^10^ Through school loudspeakers and community radio, health messages can be delivered repeatedly and creatively using familiar formats such as songs. This approach has the potential to reach large segments of the population at minimal cost, embedding eye health messages within cultural narratives that are more likely to be understood, remembered and acted on.

The Zanzibar Arts for Children’s Eyesight II (ZANZI-ACE II) intervention was underpinned by a Theory of Change, cocreated during the initial ZANZI-ACE workshop, recognising the interplay between knowledge, attitudes, social norms and health-seeking behaviours.^9^ As described by Chan *et al*,^9^ improving referral adherence begins by increasing awareness of eye health and vision correction through culturally-resonant, music-based messaging. By repeatedly exposing children and their families to engaging songs communicating key eye health messages, the intervention aimed to shift knowledge, foster positive attitudes toward eye care and reinforce supportive social norms.^10^ Repeated exposure was intended to overcome barriers such as forgetfulness, misinformation and stigma associated with spectacle wear. The approach drew on social learning theory,^11 12^ normalising positive behaviours through widely heard songs and reinforcement principles by embedding health messages within familiar and accepted cultural forms.

ZANZI-ACE II is a pilot trial that will deliver the song created during the original ZANZI-ACE project through both school-based and community-based channels. The aim is to reinforce consistent eye health messaging across home and school settings, directly engaging children while also raising awareness and shaping attitudes among parents. The song was cocreated with local musicians and children to ensure it is culturally relevant, engaging and accessible to people with different literacy levels. To our knowledge, this is the first initiative in Zanzibar to pilot a music-based, arts-driven approach to improving referral uptake after school vision screening.

A pilot trial reduces the risk of implementation failure, clarifies uncertainties in design and delivery and safeguards ethical and financial integrity. In doing so, it ensures that subsequent research and scale-up are built on a solid, locally relevant foundation, avoiding research wastage and maximising potential benefit to children in Zanzibar and beyond.^13^ This paper reports on the findings from the ZANZI-ACE II pilot trial to assess the preliminary effectiveness and cost-effectiveness of broadcast of musical performances in schools and community on screening and service update. In addition, a parallel evaluation was undertaken to assess intervention intentionality (acceptability, feasibility, adoption), efficacy (impact of the content of the pieces to address sociocultural barriers) and responsiveness (how heritage and current musical forms contributed to the interventions’ efficacy and sustainability); and the impact on children’s and parents’ knowledge, attitude and practice (KAP). These findings will be reported separately.

## Methods

### Study design and setting

We conducted a pilot, randomised interventional study with a mixed-methods evaluation between January 2024 and November 2024 in Zanzibar, covering both Unguja and Pemba islands. The 6-month postintervention follow-up period is similar to previous school-based studies.^14^,^16^ The study was informed by the ZANZI-ACE Theory of Change.^9^

### Patient and public involvement

The intervention was developed collaboratively with community members, including children, parents, teachers, musicians and local leaders, through a participatory cocreation process. Community stakeholders contributed to the design of the broadcast content to ensure cultural relevance and acceptability. The findings were disseminated to the stakeholders and community in June 2025.

### Ethics approval

The study protocol was also registered on ClinicalTrials.gov (NCT06469697). Consent was obtained through an opt-out (passive) process: information sheets were sent home with all children, and parents/guardians could decline participation by returning a signed form. All materials (information sheets, consent forms, surveys, songs) were provided in Kiswahili. For caregivers with limited literacy, trained fieldworkers read information aloud and recorded responses, ensuring equitable participation across settings. Consent covered their child’s involvement in data collection activities (vision screening, referral tracking, demographic surveys). The musical broadcasts formed part of a school-wide and community-wide public health education campaign, and therefore all children were exposed regardless of enrolment status.

### Intervention

**Figure 1:**
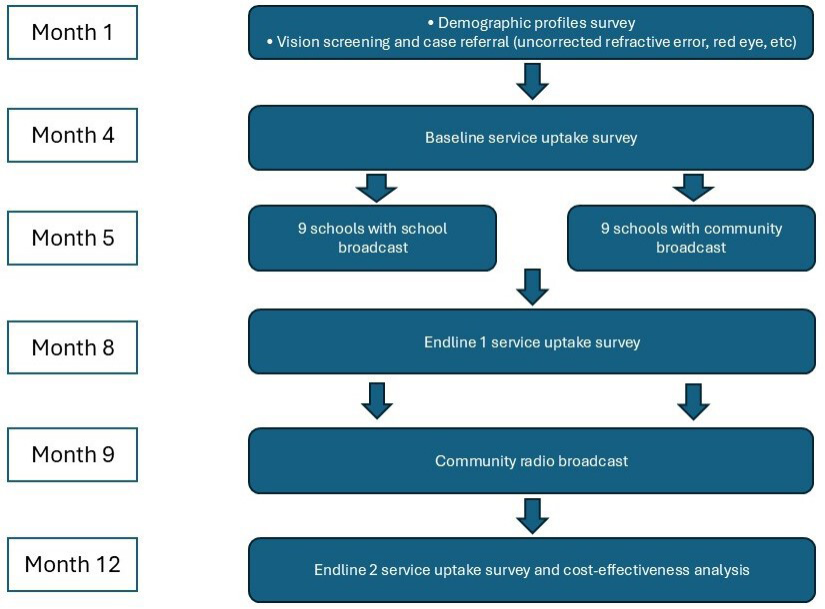
Flowchart and timeline for the study Timeline of study procedures from screening to follow-up. Children were screened at baseline (Month 1) with demographic data collected. Referral adherence was measured at baseline (Month 4), after the school broadcast (Endline 1, Month 8) and after the community broadcast (Endline 2, Month 12). School broadcasts were delivered in nine schools (Group 1), while nine schools served as the community-only group (Group 2).

The development of the ZANZI-ACE intervention has already been described in detail previously.^9^ The intervention consisted of two phases (figure 1). Phase 1 involved 3 months of school-based broadcasts, where three culturally-tailored songs (3–6 min each) were played through school loudspeakers three times a day (morning assembly, recess and school dismissal) on Mondays and Fridays. Songs broadcast at the schools were cocreated with children and teachers to ensure appropriateness for school-going children.

Phase 2 extended to three additional songs to the broader community through daily broadcasts via a local radio station over a 3-month period. The additional songs were cocreated with local musicians and community members to embed key eye health messages within familiar and engaging formats. The intervention targeted both children and parents by reinforcing consistent messaging across educational and domestic environments.

All songs were 3–6 min long, reflecting local music formats cocreated with musicians and teachers. Broadcast frequency (three times daily in schools; three times daily on radio) was determined in line with prior evidence that repeated exposure improves message retention.

### Sample, sampling, randomisation and masking

Our study design is informed by a similar study in Vietnam.^16^ We aimed to screen approximately 16 000 children to reach a sample size of 400 schoolchildren with visual acuity (VA)≤6/12 or visible eye disease (1:1 Group 1-to-Group 2 children allocation ratio), based on 80% power, a 5% significance level, a meaningful difference of a 30% increase in uptake in each group at Endline 2 (assessment after the community broadcast phase), eye morbidities prevalence of 2.5% and 5% among primary and secondary schoolchildren, respectively, and a 10% attrition rate. Teachers conducted vision screening using a Snellen chart at 6 m and torch examination for visible ocular abnormalities. The referral cut-off of 6/12 or worse is consistent with WHO/International Agency for the Prevention of Blindness recommendations for school eye health programmes.^17^ Children were eligible if they presented with VA of 6/12 or worse in either eye or had visible eye disease. We recruited five primary and four secondary schools on the Unguja and Pemba islands of Zanzibar (number of schools=18). Primary and secondary schools were eligible if they had a school-going rate of approximately 75%, a balanced gender distribution and were located within 5 km of an eye clinic.

Because national radio could not selectively broadcast to specific communities, cluster randomisation at community level was not feasible. Schools were therefore used as the unit of randomisation. The study statistician randomly selected nine schools into Group 1 (where school and community broadcasts were implemented) and another nine into Group 2, (where only community broadcasts were implemented), before any vision screening activities commenced. Despite randomising schools evenly, differing school sizes led to an approximate 1:2 ratio of referred children between Group 1 and Group 2, which resulted in larger enrolment numbers in Group 2 schools. Allocation concealment was not possible as schools were randomised in advance. Blinding of participants and outcome assessors was also not feasible, since group assignment and exposure to broadcasts were visible within schools and communities. Both Unguja and Pemba schools were represented in each group (figure 1).

The primary outcome was adherence to eye care referrals, defined as the proportion of referred children who attended a hospital-based eye examination following screening. The secondary outcome is the cost-effectiveness of the interventions, defined as cost per child reached and cost per child who accessed eye care for each intervention and combined interventions.

### Data collection

54 trained teachers (3 per school) screened approximately 16 000 children across Group 1 and Group 2 schools. Children who failed vision screening were referred for free ocular management at local eye clinics, and referral information was recorded in eye health registers maintained by teachers. Free ocular management included the initial eye examination, provision of spectacles and eye drops when indicated. Surgical referrals were outside the project scope and not covered; transport costs were not reimbursed. Referral adherence was verified using school referral registers, clinical attendance records (access granted by the Ministry of Health) and a coupon system, whereby each referred child presented a coupon at the clinic, which was cross-checked with school records.

Prior to intervention rollout, baseline data were collected from the referred children. In this manuscript, surveys were limited to the collection of baseline demographic information from referred children. Parental demographics were collected via take-home forms. Additional surveys on parental KAPs were conducted as part of a parallel evaluation and will be reported separately as per the prespecified analysis plan. Children referred for further management were followed up at Month 4 to determine baseline referral adherence. From Month 5, intervention schools began broadcasting the music pieces, and Endline 1 (assessment after the school broadcast phase) data were collected at Month 8, through verification of referral adherence via health registers and coupon tracking.

**Figure 2:**
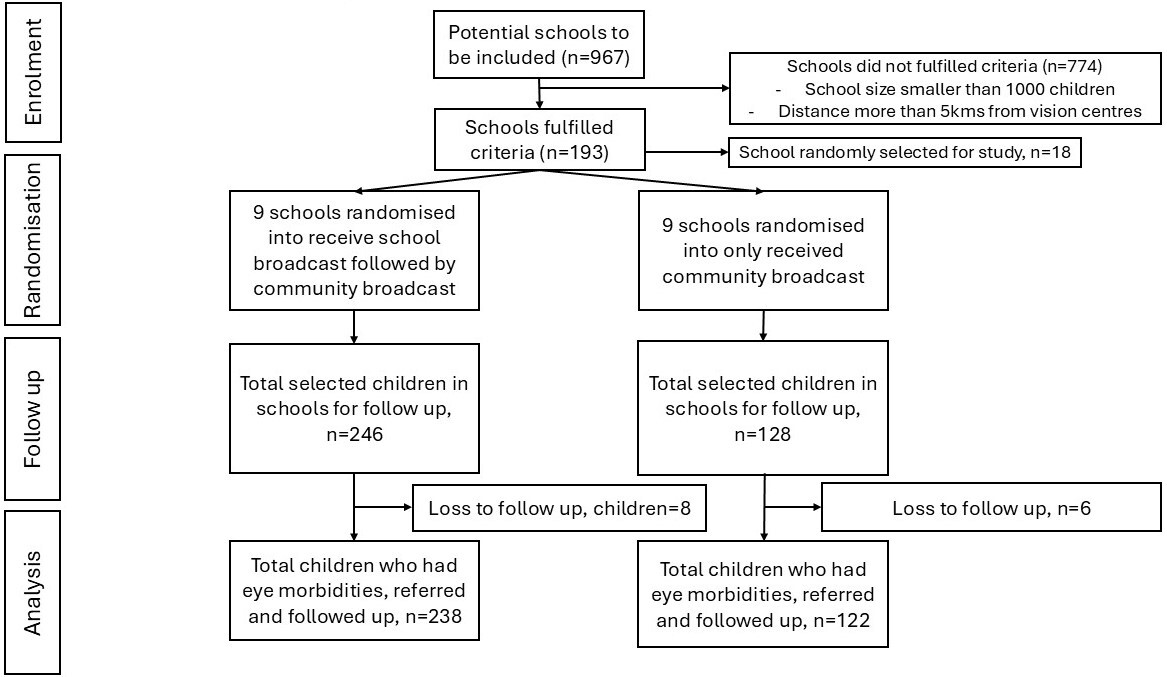
CONSORT diagram trial profile showing school eligibility, randomisation and participant flow. Of 967 schools screened for eligibility, 193 met inclusion criteria and 18 were randomly selected. Nine schools were allocated to receive school broadcasts followed by community broadcasts (Group 1), and nine schools to community broadcasts only (Group 2). Follow-up numbers and losses are shown at each stage. CONSORT, Consolidated Standards of Reporting Trials.

At Month 9, the community broadcast phase commenced, with music pieces aired three times daily on a popular local radio station. The radio station (Elimu FM) was chosen based on stakeholder feedback identifying it as the most widely listened-to education station. At Endline 2, the same cohort was reassessed at Month 12, comprising a final check of referral adherence. At each follow-up (Months 4, 8 and 12), data were also collected on children who did not attend follow-up care, and reasons for non-attendance were documented through interviews (figure 2). All data pertaining to this study can be accessed online.^18^

Furthermore, we also documented:

1. Reporting of adverse events.
2. Reporting of contamination.
  - Whether children from Group 1 schools reported, discussed or sung the songs with siblings, cousins or friends in Group 2 schools.
  - Whether a child may attend a Group 2 school but live in a household with someone attending a Group 1 school (eg, an older or younger sibling).
  - Whether enthusiastic educators or headteachers in Group 2 schools may have heard about the intervention and started replicating the songs informally.

Quantitative data were analysed using SPSS (V.27). Descriptive statistics summarised demographics and referral adherence rates. We conducted three separate difference-in-difference (DiD) analyses, always comparing the same two groups (Group 1: school+community broadcasts; Group 2: community broadcasts only). First, we assessed the effect of the school broadcast phase by comparing changes in referral adherence from baseline to Endline 1 between groups. Second, we assessed the effect of the community broadcast phase by comparing changes from Endline 1 to Endline 2, noting that both groups were exposed to community broadcasts but only Group 1 had received prior school exposure. Third, we assessed the combined effect by comparing overall changes from baseline to Endline 2, which reflects school plus community exposure (Group 1) versus community only (Group 2). These analyses did not involve a third study arm; rather, they capture different stages of exposure within the two-group design.

We reported two types of estimates: absolute percentage point changes using DiD calculations and effect sizes to assess the magnitude of change. DiD was calculated as the change in referral adherence in Group 1 before and after each intervention phase, minus the corresponding change in Group 2. Effect sizes were calculated using Cohen’s h for comparing proportions of referred children who complied with follow-up between groups, defined as h=2 arcsin(√p₁) – 2 arcsin(√p₂), where p₁ and p₂ are the proportions in Group 1 and Group 2, respectively. This measure is appropriate for binary outcomes like service uptake and allows comparison of intervention strength across phases.

These estimates serve complementary purposes: DiD captures raw percentage differences, while effect sizes help interpret the strength of associations independent of sample size. Subgroup analyses examined differences by sex, age group and geographical location. All statistical analyses were considered exploratory due to the pilot nature of the study. A definitive trial was deemed warranted if referral adherence improved by ≥25%, considering a meaningful threshold in school eye health. The trial was originally registered to include up to 1295 participants, comprising both children and adults, for primary and secondary analyses. This manuscript reports on a prespecified subset of child participants, who were the target population for the primary analysis of this pilot study. Adult participants, included for secondary outcomes, will be reported separately.

### Cost-effectiveness and sensitivity analysis

Spending for all cost items was collected on a real-time basis. Costs were calculated from the provider (organiser) perspective, including programme development, teacher time and radio airtime. Families bore additional costs such as transport and surgery, which were not included. Costs were categorised into those related to development of strategy (ie, cocreation workshops, recording of musical pieces), preparation for implementation (ie, training of teachers, teacher’ time) and delivery of strategy (ie, speakers, payment for radio broadcasts). To assess the cost-effectiveness of the school and community broadcast interventions, we calculated cost per child accessing care (calculated using the number of referred children who attended services), and cost per person reached (included the wider population exposed to the broadcasts). The cost per child reached was obtained by dividing the total cost of implementing each intervention by the estimated number of individuals exposed to the intervention. Since the school broadcast had a clearly defined audience of 480 children, direct exposure could be determined. In contrast, community broadcast exposure was estimated based on radio listenership data, approximating an audience of 49 813 adults. Radio exposure estimates were obtained from Zanzibar Broadcasting Authority surveys; individual-level exposure could not be measured. The combined intervention reach was set at 500 000, accounting for possible overlaps. For the cost per child who accessed care, we calculated based on marginal intervention costs directly related to service uptake (eg, transport fees and treatment costs, such as spectacles and eyedrops), not including one-time development or setup costs. We divided the total cost of each intervention by the number of children who sought eye care after the intervention, as determined from study data: 61 out of 149 for the school broadcast, 25 out of 65 for the community broadcast and 104 out of 149 for the combined approach.

Although registries were the main source, variation in record completeness and coupon returns could lead to underestimation/overestimation. Sensitivity analysis was conducted to account for this uncertainty by modelling plausible ranges. We adjusted the estimated number of children who sought care by ±20% to evaluate how fluctuations in uptake affected cost-effectiveness. The adjusted estimates allowed for three scenarios: a worst-case (80% of the base case), a base case (100%) and a best-case (120%). For each scenario, the new cost per child who accessed care was recalculated to assess the robustness of the cost-effectiveness estimates under different assumptions. This approach provided insight into how resilient each intervention was to changes in actual service uptake and helped identify which intervention remained cost-effective even if fewer children accessed services. A definitive trial is suggested if we achieve feasible delivery at <US$5 per child, aligning with benchmarks used for cost-effectiveness in low- and middle-income country (LMIC) school eye health programmes in Sub-Saharan Africa (SSA) of between US$1 and US$5.^19^ Reporting is completed per the CONSORT (Consolidated Standards of Reporting Trials) 2010 Extension for Pilot and Feasibility Trials Checklist.

## Results

### Participant characteristics

A total of 374 children with reported eye problems were included in the study, with 246 in Group 1 and 128 in Group 2 (table 1). The distribution of sex was similar between groups (p=0.858), but a higher proportion of children from Pemba (rural areas) were Group 1 compared with Group 2 (p=0.008). The full child age range was 6–18 years, with mean age comparable between groups (13.92 vs 13.64 years, p=0.229) (table 1). Of the 246 children in Group 1, 238 (96.7%) were successfully followed up, while of the 128 children in Group 2, 122 (95.3%) were followed up and included in the analysis.

**Table 1:** Characteristics of children included in the study, N=374

Characteristics	Group 1	Group 2	P value
	Children at baseline	Children at baseline	
	n=246 (65.8%)	n=128 (34.2%)	
Sex			
Male	67 (27.2%)	33 (25.8%)	0.858*
Female	179 (72.8%)	95 (74.2%)	
Island			
Pemba (Rural)	129 (52.4%)	48 (37.5%)	0.008*
Unguja (Semiurban)	117 (47.6%)	80 (62.5%)	
Age group			
9 and younger	28 (11.4%)	22 (17.2%)	0.229†
10 to 12 years old	50 (20.3%)	18 (14.1%)	
13 to 16 years old	117 (47.6%)	58 (45.3%)	
17 and older	51 (20.7%)	30 (23.4%)	
Mean age±SD	13.92±3.417 years	13.64±3.267 years	0.449‡
Education level			
No formal education	59 (24.2%)	34 (26.2%)	0.054
Primary education	58 (23.7%)	29 (22.7%)	
Secondary education	103 (41.7%)	53 (41.4%)	
Higher than secondary education	26 (10.4%)	12 (9.7%)	
Occupation			
Agriculture and fishery	115 (46.9%)	64 (49.9%)	0.011
Business	67 (27.2%)	31 (23.4%)	
Professional	28 (11.3%)	15 (11.8%)	
Housewife	19 (7.8%)	11 (8.8%)	
Masonry	9 (3.6%)	4 (3.6%)	
Normal physical labour	5 (2.0%)	3 (2.4%)	
Retired	2 (0.7%)	0 (0%)	
Student	1 (0.4%)	0 (0%)	
Total	246 (100%)	128 (100%)	

Group 1=3 months of school broadcast followed by community broadcast for 3 months.

Group 2=community broadcast only at the same time as community broadcast in Group 1.

* Continuity correction.

† Pearson χ².

‡ Independent sample t-test.

### Referral adherence rates

At baseline, referral adherence rates were similar between groups, with 42.6% in Group 2 and 37.4% in Group 1 (p=0.397). Following the 3-month school broadcast intervention (Endline 1), the referral adherence rate increased significantly to 40.9% in Group 1, compared with 7.1% in Group 2 (p<0.0001). The adjusted effect size of the school broadcast alone was 0.256 (95% CI: 0.169 to 0.343), reflecting a substantial positive impact (table 2).

**Table 2:** Referral adherence rate, difference-in-difference and effect sizes of school broadcast, community broadcast and, school and community broadcast

	Baseline		Endline 1		Endline 2		School and community broadcast	
	Group 2	Group 1	Group 2	Group 1	Group 2	Group 1	Before	After
Compliance rate	42.6%	37.4%	7.1%	40.9%	38.5%	48.9%	42.9%	69.8%
Difference in difference, %			−35.5%	+3.5%*	+31.3%	+7.9%†	+24.6%	+43.7%‡
Effect size (95% CI)			0.26 (0.17 to 0.34)		0.21 (0.08 to 0.33)		0.44 (0.36 to 0.52)	

Group 1=3 months of school broadcast followed by community broadcast for 3 months.

Group 2=community broadcast only at the same time as community broadcast in Group 1.

Endline 1=assessment after the school broadcast phase.

Endline 2=assessment after the community broadcast phase.

* p<0.0001.

† p=0.265.

‡ p=0.0006.

After the additional 3-month community radio broadcast (Endline 2), service uptake improved modestly, reaching 48.9% in the Group 1 versus 38.5% in Group 2 (p=0.265). The adjusted effect size for the community broadcast alone was 0.205 (95% CI: 0.083 to 0.326), suggesting a moderate but statistically non-significant added effect.

When combining the school and community broadcast interventions, 69.8% of children in Group 1 eventually attended an eye clinic, compared with 42.9% in Group 2 (p=0.0006). The overall adjusted effect size for the combined intervention was 0.437 (95% CI: 0.358 to 0.516), demonstrating a strong cumulative impact on improving referral adherence. (table 2)

### Subgroup analyses

Sex distribution remained similar between groups across time points. However, geographical differences emerged: by Endline 2, a higher proportion of children from Pemba accessed care in Group 1 compared with Group 2 (p=0.030), suggesting improved access among rural populations. Age distribution of attendees was broadly similar, with most children who attended care aged 10 years or older (p=0.352).

### Reasons for non-compliance

Among children who did not comply with referrals, the most common reasons provided were not receiving the referral letter (31%), caregivers lacking time (24%) and caregivers perceiving no need for follow-up (22%). Spectacle compliance was low in both groups, with 33% adherence in Group 2 and 37% in Group 1.

### Adverse events and contamination

No adverse events or unintended effects were observed or reported in either group throughout the intervention period. The musical broadcasts were well-tolerated by children, teachers and community members, with no instances of distress, problems or technical issues requiring intervention. There was no indication that Group 2 participants had been exposed to the musical content in Group 1 from Month 4 to Month 7. This is likely because siblings usually attend the same school, and there was limited peer contact across groups. As such, the risk of contamination appears minimal.

### Cost-effectiveness of the interventions

The cost-effectiveness analysis showed that the school broadcast intervention resulted in a cost of US$4.65 per child who accessed care, while the community broadcast cost US$3.11 per child and the combined intervention had the lowest cost at US$3.02 per child. Although the community intervention reached the largest audience (49 813 people) at the lowest cost per person reached (US$0.29), the school broadcast was more effective in prompting individual service uptake. The combined intervention reached 50 100 people at a cost of US$0.37 per person. Sensitivity analysis revealed that the school broadcast intervention remained cost-efficient across different uptake scenarios, with the cost per child who accessed care ranging from US$5.79 (80% of base case uptake) to US$3.89 (120%). For the community broadcast, costs varied from US$3.89 (80%) to US$2.59 (120%). The combined intervention consistently showed the greatest efficiency across all scenarios, with costs per child accessing care ranging from US$3.78 (80%) to US$2.51 (120%) (table 3).

**Table 3:** Cost-effectiveness analysis and sensitivity analysis

Cost items	School broadcast	Community broadcast	School and community broadcast
Costs-effectiveness analysis			
Development of strategy (USD)	12 795	12 795	12 795
Preparation for implementation (USD)	3096	198	3094
Delivery of strategy (USD)	1254	1250	2504
Total (USD)	17 145	14 243	18 593
People reached (n)	9012	49 813	50 100
Cost per person reached (USD)	1.91	0.29	0.37
Cost to access services (USD)	283.65	77.75	314.08
Referral adherence rate	61/149	25/65	104/149
Cost per referred child accessed service (USD)	4.65	3.11	3.02
Sensitivity analysis			
80% of base case			
Adjusted children accessed care (n)	49	20	83
Adjusted cost/referred child accessing care (USD)	5.79	3.89	3.78
100% of base case			
Adjusted children accessed care (n)	61	25	104
Adjusted cost/referred child accessing care (USD)	4.65	3.11	3.02
120% of base case			
Adjusted children accessed care (n)	73	30	125
Adjusted cost/referred child accessing care (USD)	3.89	2.59	2.51

## Discussion

This pilot study provides preliminary evidence that a culturally tailored school and community broadcast intervention may improve eye care referral adherence among schoolchildren in Zanzibar. Improvements were most notable following the school broadcast phase, with additional, more modest gains after community broadcasting. While the school-based intervention was more impactful in increasing referral adherence, the community broadcast and combined approach were more cost-effective per child who accessed care. These findings support the potential for a layered communication strategy and signal readiness to proceed to a fully powered definitive trial.

Although we initially aimed for a 1:1 allocation of referred children between groups, logistical constraints and school-level enrolment variation resulted in a 1:2 ratio, with more referred children in Group 2. This imbalance may affect statistical precision, potentially widening CIs in Group 1 and influencing the comparability of group-level estimates. However, as this was a pilot trial, the primary aim was to assess feasibility and preliminary signals of effectiveness, rather than to draw definitive conclusions.

At baseline, referral adherence rates were similar between groups, consistent with findings from other LMIC settings.^2^,^4^ After the school broadcast intervention, referral adherence increased substantially, suggesting that repeated, culturally tailored school messaging can influence health-seeking behaviour.

Community broadcasting appeared to add modest further gains but was more impactful when layered on top of school messaging alone, aligning with prior evidence that mass media interventions often require interactive reinforcement to maximise behaviour change.^10^ Combined school and community exposure yielded the highest service uptake rates, supporting multilevel, cross-setting approaches to health promotion.^19^

While community broadcasts reached a much larger audience at a lower cost per person (US$0.29), school broadcasts resulted in a slightly higher proportion of children accessing services. However, it was the combined school and community intervention that produced the highest overall referral compliance—104 out of 149 referred children accessed care (69.8%). This highlights the value of layering communication channels to reinforce messages and drive behaviour change.

Although school broadcasts had a stronger standalone impact on service uptake (61 children), they were the most expensive per child who accessed care (US$4.65). In contrast, community and combined interventions were more cost-efficient at US$3.11 and US$3.02 per child, respectively. Sensitivity analyses confirmed that the combined intervention remained the most cost-effective across all uptake scenarios, with adjusted costs per child ranging from US$3.78 (at 80% uptake) to US$2.51 (at 120%). These findings suggest that while school-based messaging can improve behavioural response, combining it with broader-reaching community broadcasts offers the most effective and efficient strategy for increasing referral compliance in eye health programmes. As anticipated for a pilot study, the primary goal was to gather early signals of effectiveness, rather than to draw definitive conclusions. The findings nonetheless provide valuable guidance for the design of a larger, fully powered trial.

Findings met the threshold to proceed to a definitive trial, including ≥25% increase in adherence and feasible delivery at <US$5 per child. Building on the lessons learnt, future trial designs should incorporate several key refinements to strengthen methodological rigour and maximise impact. First, stratified randomisation by key variables, such as island location and school size, should be employed to minimise baseline imbalances between study groups. Second, using the observed adherence rates from the pilot (42.9% in Group 2 vs 69.8% in Group 1), we recommend a fully powered cluster randomised trial to evaluate the broadcast intervention’s impact on referral adherence. Based on pilot adherence rates and assuming an intracluster correlation of 0.02 and 20 referred children per school, approximately 20 schools and 400 referred children would be needed to achieve sufficient power. To reach this, around 15 000–18 000 children should be screened.

This pilot study had several strengths. The intervention was cocreated with community members, ensuring strong cultural relevance and acceptability. We saw a low risk of contamination between study groups during the school broadcast phase. Despite the potential for peer or sibling sharing of intervention content, our follow-up with participants and field implementers found no evidence of crossover exposure. This may reflect the common practice of siblings attending the same school and limited cross-school interactions in the study setting. Furthermore, the absence of reported adverse events or implementation issues supports the acceptability and feasibility of using musical messaging in school settings.

However, several limitations must be acknowledged. First, as a pilot study, the sample size was modest and limits the generalisability of the findings. Differences in school size and a higher proportion of rural children in Group 1 may have introduced imbalance, potentially influencing uptake. Second, adherence tracking relied primarily on school records and self-reports from participants, which may introduce reporting bias. Three, blinding of participants and outcome assessors was not possible due to the nature of the school and community broadcast intervention, potentially introducing performance bias. Four, Group 2 experienced a longer delay from screening to intervention due to national radio scheduling, which may have reduced recall of the screening. Finally, the relatively short follow-up period constrained the ability to assess the long-term sustainability of behaviour change.

### Conclusion

This pilot study offers preliminary evidence that a culturally tailored broadcast intervention, delivered through schools and communities, can improve referral adherence among schoolchildren in Zanzibar. The greatest gains in service uptake were observed following the school-based broadcasts, while community broadcasts contributed additional, though more modest, improvements. Although the school intervention had the greatest impact on referral adherence, the combined school and community approach proved most consistently cost-effective across all scenarios. Based on these results, a fully powered definitive trial is warranted to confirm effectiveness, optimise delivery and inform policy for broader implementation.

## Data Availability

Data are available in a public, open access repository.
