# Supplementary material for: Preliminary effectiveness of musical messaging to improve child eye health service uptake in Zanzibar: a pilot randomised trial
Source: BMJ Open. 2025 Sep 23;15(9):e107348. doi: 10.1136/bmjopen-2025-107348 (PMC12458887; doi:10.1136/bmjopen-2025-107348)
Supplement: Supplementary file 1 [file bmjopen-2025-107348.reviewer_comments.pdf]

PEER REVIEW HISTORY

BMJ Open publishes all reviews undertaken for accepted manuscripts. Reviewers are asked to complete a checklist review form and are provided with free text boxes to elaborate on their assessment. These free text comments are reproduced below.

ARTICLE DETAILS

Title (Provisional)

A Pilot Randomised Trial on Preliminary Effectiveness of Musical Messaging to Improve Child Eye Health Service Uptake in Zanzibar

Authors

Omar, Fatma; Othman, Omar Juma; Yong, Ai Chee; Belluigi, Dina; Graham, Christine; Graham, Ronnie; Mashayo, Eden; Chan, Ving Fai

---

VERSION 1 - REVIEW

**Reviewer**	**1**
**Name**	**Asare , Afua**
**Affiliation**	**University of Utah Health**
**Date**	**07-Aug-2025**
**COI**	**None**

This is an important and novel study informed by implementation science methodology to assess the preliminary effectiveness of school- and community- based programming to improve uptake of child eye health services in Zanzibar. Most comments provided below are to improve clarity by expanding on some aspects of the manuscript and explaining jargon that readers may not be familiar with. Some of the additional content requested may be appropriate to include in an appendix, or with preference to a prior study (if addressed in another study by the authors). Detailed suggestions and recommendations for changes are below:

1) How was informed consent completed by parents or guardians? Did children take paper consent forms home? Was it consenting to participate, or not to participate (opt in/active or opt out/passive consent)? I would also be curious to know what proportion of eligible children received a consent form to participate (if this data is available). What study activities did parents and caregivers consent to – the screening, or the referral music intervention? If parents did not consent to participate in the study how did authors manage the music intervention broadcast? Surely, children who did not consent would have heard the broadcast too.

2) Surveys are only mentioned briefly in the methods as a tool to collect demographic data only. However, in Figure 1 there is an indication that parental knowledge attitude and practice surveys were completed as well as service uptake surveys three times during the study. Please clarify the purpose of each of these surveys as well as expand on how the surveys were distributed (did children take paper surveys home and return them later), how were they completed (in person

interviews? virtual? paper?), who completed them (parents or caregivers?), and response rate of surveys. Including a copy of the survey in the manuscript – at the very least in the appendix would also be helpful to readers?

3) Did the team experience health literacy challenges (especially those in rural areas)?. How did investigators handle this, especially as it relates to informed consent, collecting demographic data and completion of KAP and uptake surveys? with parents completing consent forms? Was the intervention provided in the local language?

4) The focus of this paper is on the referral adherence intervention, but I think readers would be curious to know what vision screening procedures/tests were conducted. A single sentence on this should be appropriate. Also in Line 154 – what is the cutoff of 6/12 based on ? Do you have literature to support the choice of this cutoff?

5) There appears to be discrepancies in how Group 1 & 2 are defined. In the abstract, group 1 and 2 are described as follows, "*Group 1 is 3 months of school broadcast followed by community broadcast for 3 months and Group 2 – community broadcast only at the same time as community broadcast in group 1*". However, in Table 2, it appears that Group 2 also received school broadcast . Please clarify. Authors should also clarify in the methods (sample, sampling… making), that schools from both Unguja and Pemba islands were represented in both groups.

6) 18 schools were assigned to group 1 & 2. Are there differences in socioeconomic status and potentially health literacy between caregivers in each group? How may this have impacted the results of the study (i.e., referral adherence). Demographic data were collected on socioeconomic status, could these be included in Table 1?

7) How or what decisions went into the research team deciding how long the musical intervention should be (why 3 to 6 minutes?) and how frequently it should be provided? (3 times a day and twice a week in schools, 3 times daily at a radio station)? Is there prior literature to support this

8) Line 150 – I assume the sample size of 400 school children represents children with poor vision (VA of 6/12 or worse)? Please include

9) Line 159-160 –What do authors mean by a community trial design here and why wasn't this feasible using national radio services? Expand on this point

10) Line 166-167 &383 ("*Allocation concealment… community broadcasting*"). Please clarify what you mean here and why allocation concealment and masking was not possible. What does blinding mean here, is it blinding of participants from each other and outcome assessors? In other words, one child not knowing that the other is in the study and a particular group? Please clarify

11) Line 177-178 - Authors mention that free ocular management was provided to children at local clinics. Please indicate the scope of the free ocular management. Did it include e.g., a first eye exam only (or ongoing follow-ups as well), transportation, treatment, spectacles, eyedrops etc. What if the child required surgery was that covered too?

12) Line 179 – What is the coupon system and how is it used to facilitate and track referrals. Please expand

13) Line 181-183 - The authors mention demographic surveys that captured a number of variables including parent demographics. If my read of this section is correct, these variables were collected from referred children. I would think that collecting data from children (especially younger children) would be problematic and lead to inaccuracies. Younger children especially may not remember their age, history of eye visits and treatments how much more parent demographics (education, etc.). Authors should also include in results the age range of children (and not average age alone).

14) Line 186 – Please expand on how referral adherence was verified using health registries and coupon tracking. Where were these health registries located? Within the health care system? If it is the health system, how did they gain access to it? Please also point to the appropriate figure that matches up with the data described here (Figure 1?)

15) Line 188 – Authors describe that they played the musical pieces on a popular local radio station. How did authors determine popularity?

16) Line 210 – I appreciate the authors taking the time to explain how they measured difference-in-difference and effect sizes. I'd recommend they qualify 'proportions' here. Is it proportions of children that complied? If so, please state this

17) Line 218 – What are the authors rationale for choosing cutoffs for the definitive trial. E.g., 25% increase in adherence and <$5 per child feasible delivery
18) Line 223 – Authors should indicate what cost perspectives they quantified here. Is it the organizers perspectives, or society (i.e. Patient perspectives). If it is organizer perspectives, did they organizers pay for transportation, treatment, spectacles and eyedrops – or were the families required to take this cost?

19) Line 231 – Did the authors collect any info on the listenership? What estimated proportion of children/caregivers heard the radio broadcasts? This could play a role in compliance

20) Line 242 – 243 – I appreciate the sensitivity analyses that were done here, but authors mention earlier that they used registries to determine adherence. Therefore I am unsure about the uncertainty that authors describe here.

21) Line 258-259: Please clarify what the control and intervention groups are here. If group 1 and 2 please specify. An expanded description of these groups is also needed in the methods section.

**Reviewer**	**2**
**Name**	**Summers, Allison**
**Affiliation**	**Oregon Health & Science University**
**Date**	**19-Aug-2025**
**COI**	**None**

This is an interesting and valuable study that could serve as an example for other public health intervention campaigns. Work is needed to clarify the manuscript.

I suggest a more compelling title. Drop the 1st sentence

Line 66: define “vertical and integrated models”

What is “Endline 2” or “Endline 1?”-- Line 152, 185, 188 and throughout the manuscript. Define

Rename the groups to reflect their definition to reduce confusion; instead of Group 1 and Group 2, perhaps “School-Community Group” and “Community-Only Group.” In line 177 and 258-259, you refer to the groups as intervention and control schools, then 363 you refer to study arms. Use consistent terminology throughout the manuscript.

172-173—should be cost per referred child who was reached. It is confusing throughout the manuscript if you are discussing the total number of children who heard the message, or the total TARGET number, which would be those referred. If listing the total number who heard the message, use some space in the discussion to justify this by addressing the benefit of public health awareness beyond the children referred (eg better attitudes towards glasses wear to support those who need them)

Do not list where to insert the figures

Line 239-240 is confusing—how do you know who sought care after only the school broadcast? Isn’t this the same children as the combined group, perhaps before the community broadcast? The other portions of the manuscript discuss the 2 groups, but you have divided into 3 groups here.

Table 3 labels are not consistently or clearly named

the abstract is confusing. Don’t mention beyond that included in this manuscript. Clarify the intervention.

Figure 2 numbers don’t seem to match the text:

238 of 246 followed up (implies they were referred and received care) would be a 96.7% uptake, and 122 of 128 would be 95.3%

discussion

The difference in school size in Group 1 compared to Group 2 may have led to bias as a larger school may be in a larger community, with different healthcare attitudes and behavior. In a separate area, the authors state a higher proportion of rural children in the intervention group, although it is unclear if these are the same schools (large schools in

a rural area with large catchment area, vs some large semiurban schools and some rural schools)

Line 337—I believe you meant “more impactful”? otherwise this contradicts elsewhere

Line 372—analysis of parent and child engagement is not in this manuscript, but rather whether they followed through with seeking care.

Groups 1 and 2 are not balanced in that the time from screening to the first intervention was much longer in Group 2, which would result in the parent and child to be less likely to remember the failure and connect it with the content. This may not be adjustable due to the national broadcast, but it’s effect should be acknowledged

---

VERSION 1 - AUTHOR RESPONSE

Reviewer 1:

1. How was informed consent completed by parents or guardians? Did children take paper consent forms home? Was it consenting to participate, or not to participate (opt in/active or opt out/passive consent)? I would also be curious to know what proportion of eligible children received a consent form to participate (if this data is available). What study activities did parents and caregivers consent to – the screening, or the referral music intervention? If parents did not consent to participate in the study how did authors manage the music intervention broadcast? Surely, children who did not consent would have heard the broadcast too.

Response: Consent was opt-out (passive consent): written information sheets were sent home with all children, and parents/guardians could decline their child’s participation by returning a signed form. Parents and caregivers were consenting to their child’s participation in data collection activities (vision screening, referral tracking, and demographic surveys). The musical broadcasts were delivered as a school- and community-wide public health education activity, and therefore all children were exposed to the intervention regardless of enrolment. This approach was approved by the Zanzibar Health Research Institute and Queen’s University Belfast. We revised this in LINE 441 - 455

2. Surveys are only mentioned briefly in the methods as a tool to collect demographic data only. However, in Figure 1 there is an indication that parental knowledge attitude and practice surveys were completed as well as service uptake surveys three times during the study. Please clarify the purpose of each of these surveys as well as expand on how the surveys were distributed (did children take paper surveys home and return them later), how were they completed (in person interviews? virtual? paper?), who completed them (parents or caregivers?), and

response rate of surveys. Including a copy of the survey in the manuscript – at the very least in the appendix would also be helpful to readers?

Response: In this manuscript we report only on referral adherence outcomes. While children and parental knowledge, attitude, and practice (KAP) and engagement surveys were conducted as part of a parallel evaluation, these will be reported separately in forthcoming manuscripts as per our pre-specified analysis plan. Accordingly, we revised the Methods – Data Collection section to specify that only demographic data and referral adherence outcomes are presented here, and we streamlined Figure 1 to show only processes relevant to this manuscript. To maintain transparency, we have cross-referenced the forthcoming KAP/QOL manuscripts (LINE 192–197) and indicated that the full survey instruments will be published there. Copies are available upon request if required.

3: Did the team experience health literacy challenges (especially those in rural areas)? How did investigators handle this, especially as it relates to informed consent, collecting demographic data and completion of KAP and uptake surveys? with parents completing consent forms? Was the intervention provided in the local language? Did the team experience health literacy challenges (especially those in rural areas)? How did investigators handle this… Was the intervention provided in the local language?

Response: All materials (consent forms, surveys, and songs) were in Kiswahili. Fieldworkers assisted illiterate caregivers by reading information aloud and recording responses. This ensured equitable participation. We added detail in Methods LINE 441-447.

4: The focus of this paper is on the referral adherence intervention, but I think readers would be curious to know what vision screening procedures/tests were conducted. A single sentence on this should be appropriate. Also in Line 154 – what is the cutoff of 6/12 based on ? Do you have literature to support the choice of this cutoff?

Response: Teachers screened children using Snellen charts at 6 metres and torch examination for ocular abnormalities. The 6/12 cutoff follows WHO/IAPB recommendations for school eye health and prior SSA studies. We added in Methods – Data Collection (LINE 156-159) with supporting citation.

5: There appears to be discrepancies in how Group 1 & 2 are defined. In the abstract, group 1 and 2 are described as follows, “Group 1 is 3 months of school broadcast followed by community broadcast for 3 months and Group 2 –

community broadcast only at the same time as community broadcast in group 1". However, in Table 2, it appears that Group 2 also received school broadcast. Please clarify. Authors should also clarify in the methods (sample, sampling... making), that schools from both Unguja and Pemba islands were represented in both groups Discrepancies in how Group 1 and 2 are defined... Please clarify.

Response: We clarified that Group 1 = 3 months of school broadcast followed by community broadcast for 3 months and Group 2 = community broadcast only at the same time as community broadcast in Group 1. Both Unguja and Pemba schools were represented in each group (LINE 174).

To clarify, Group 2 does not receive school broadcasts. We conducted three separate difference-in-difference (DiD) analyses, always comparing the same two groups (Group 1 and Group 2. First, we assessed the effect of the school broadcast phase by comparing changes in referral adherence from baseline to Endline 1 between groups. Second, we assessed the effect of the community broadcast phase by comparing changes from Endline 1 to Endline 2, noting that both groups were exposed to community broadcasts but only Group 1 had received prior school exposure. Third, we assessed the combined effect by comparing overall changes from baseline to Endline 2, which reflects school plus community exposure (Group 1) versus community only (Group 2). These analyses did not involve a third study arm; rather, they capture different stages of exposure within the two-group design. We added this information in Methods (LINE 222 – 232)

6. 18 schools were assigned to group 1 & 2. Are there differences in socioeconomic status and potentially health literacy between caregivers in each group? How may this have impacted the results of the study (i.e., referral adherence). Demographic data were collected on socioeconomic status, could these be included in Table 1?

Response: No significant differences were found in parental education or employment between groups. We agree SES may influence adherence and have included available data. We updated Table 1 with SES indicators.

7. How or what decisions went into the research team deciding how long the musical intervention should be (why 3 to 6 minutes?) and how frequently it should be provided? (3 times a day and twice a week in schools, 3 times daily at a radio station)? Is there prior literature to support this?

Response: Length was co-determined with musicians/teachers to reflect local song formats. Repetition (3x daily) followed evidence from prior mass-media interventions (Wakefield et al.). We added rationale in Methods – Intervention. (LINE 146-149)

8. Line 150 – I assume the sample size of 400 school children represents children with poor vision (VA of 6/12 or worse)? Please include

Response: Yes, the 400 target referred specifically to children with VA ⩽6/12 or visible eye disease. We clarified in Methods – Sampling. (LINE 151-152)

9. Line 159-160 –What do authors mean by a community trial design here and why wasn't this feasible using national radio services? Expand on this point

Response: Because national radio could not selectively broadcast to specific communities, cluster randomisation at community level was not feasible. Schools were therefore used as the unit of randomisation. We clarified in Methods – Sampling and Randomisation (LINE 164-166).

10. Line 166-167 &383 ("Allocation concealment… community broadcasting"). Please clarify what you mean here and why allocation concealment and masking was not possible. What does blinding mean here, is it blinding of participants from each other and outcome assessors? In other words, one child not knowing that the other is in the study and a particular group? Please clarify.

Response: Allocation concealment was not possible as schools were randomised in advance. Blinding of participants and outcome assessors was also not feasible, since group assignment and exposure to broadcasts were visible within schools and communities. We revised Methods – Sampling and Randomisation (LINE 171-174).

11. Line 177-178 - Authors mention that free ocular management was provided to children at local clinics. Please indicate the scope of the free ocular management. Did it include e.g., a first eye exam only (or ongoing follow-ups as well), transportation, treatment, spectacles, eyedrops etc. What if the child required surgery was that covered too?

Response: Free management included initial examination, spectacles, and eye drops. Surgical referrals were outside project scope and not covered. Transport was not reimbursed. We clarified in Methods – Data Collection (LINE 186-188).

12. Line 179 – What is the coupon system and how is it used to facilitate and track referrals. Please expand.

Response: Each referred child received a coupon. Clinics collected these at attendance, enabling cross-check with school registers to verify referral adherence. We expanded description in Methods – Data Collection (LINE 200-201).

13. Line 181-183 - The authors mention demographic surveys that captured a number of variables including parent demographics. If my read of this section is correct, these variables were collected from referred children. I would think that collecting data from children (especially younger children) would be problematic and lead to inaccuracies. Younger children especially may not remember their age, history of eye visits and treatments how much more parent demographics (education, etc.). Authors should also include in results the age range of children (and not average age alone).

Response: Parent demographics were collected from the parental survey, not from children. We clarified this LINE 192/195. The full child age range (6–18 years) is now reported in Results LINE 293-294.

14. Line 186 – Please expand on how referral adherence was verified using health registries and coupon tracking. Where were these health registries located? Within the health care system? If it is the health system, how did they gain access to it? Please also point to the appropriate figure that matches up with the data described here (Figure 1?)

Response: Adherence was verified via school registers, clinic registers, and coupon returns. Access to health registers was approved by MoH. We clarified in Methods – Data Collection (LINE 188-191).

15. Line 188 – Authors describe that they played the musical pieces on a popular local radio station.

Response: The radio station (Elimu FM) was chosen based on stakeholder feedback identifying it as the most widely listened-to education station. We added explanation in Methods – Intervention (LINE 203-204).

16. Line 210 – I appreciate the authors taking the time to explain how they measured difference-indifference and effect sizes. I'd recommend they qualify

'proportions' here. Is it proportions of children that complied? If so, please state this.

Response: Proportions refer to the percentage of referred children who complied with referral. We clarified in Methods – Analysis (LINE 234 – 235)

17. Line 218 – What are the authors rationale for choosing cutoffs for the definitive trial. E.g., 25% increase in adherence and <$5 per child feasible delivery. Rationale for definitive trial cutoffs (≥25% adherence, <$5 per child).

Response: Thresholds were set a priori: ⩾25% improvement reflects a clinically meaningful change seen in prior school eye health interventions (LINE 244-246), and <$5 per child aligns with benchmarks used for cost-effectiveness in LMIC school health programmes (LINE 285-286).

18. Line 223 – Authors should indicate what cost perspectives they quantified here. Is it the organizers perspectives, or society (i.e. Patient perspectives). If it is organizer perspectives, did they organizers pay for transportation, treatment, spectacles and eyedrops – or were the families required to take this cost.

Response: Costs were calculated from the provider (organiser) perspective, covering programme development, teacher time, and radio airtime. Families bore additional costs (transport, surgery). We specified in Methods – Cost-effectiveness (LINE 252-254).

19. Line 231 – Did the authors collect any info on the listenership? What estimated proportion of children/caregivers heard the radio broadcasts? This could play a role in compliance.

Response: We relied on Zanzibar Broadcasting Authority estimates (~49,813 regular listeners). We did not collect individual-level exposure data. We added clarification in Methods – Cost-effectiveness (LINE 265–267).

20. Line 242 – 243 – I appreciate the sensitivity analyses that were done here, but authors mention earlier that they used registries to determine adherence. Therefore I am unsure about the uncertainty that authors describe here.

Response: Although registries were the main source, variation in record completeness and coupon returns could lead to under/over-estimation. Sensitivity analysis accounted

for this uncertainty by modelling plausible ranges. We clarified in Methods – Cost-effectiveness and Sensitivity Analysis (LINE 275-277).

21. Line 258-259: Please clarify what the control and intervention groups are here. If group 1 and 2 please specify. An expanded description of these groups is also needed in the methods section.

Response: We clarified that Group 1 = 3 months of school broadcast followed by community broadcast for 3 months and Group 2 = community broadcast only at the same time as community broadcast in Group 1. These terms are now used consistently throughout the manuscript, including abstract, methods, tables, figures, and discussion.

Reviewer 2

1. I suggest a more compelling title. Drop the 1st sentence。

Response: We have revised the title to be more concise and compelling by removing the first sentence.

2. Line 66 – Define "vertical and integrated models."

Response: We added definitions at first mention. Vertical models refer to vertical models (stand-alone school eye health programmes) and integrated models (delivered alongside school feeding).

3. What is "Endline 2" or "Endline 1?"-- Line 152, 185, 188 and throughout the manuscript. Define

Response: We now define Endline 1 as the assessment after the school broadcast phase, and Endline 2 as the assessment after the community broadcast phase. This terminology is used consistently.

4. **Rename the groups to reflect their definition to reduce confusion; instead of Group 1 and Group 2, perhaps "School-Community Group" and "Community-Only Group." In line 177 and 258-259, you refer to the groups as intervention and control schools, then 363 you refer to study arms. Use consistent terminology throughout the manuscript.**

Response: We clarified that Group 1 = 3 months of school broadcast followed by community broadcast for 3 months and Group 2 = community broadcast only at the

same time as community broadcast in Group 1. These terms are now used consistently throughout the manuscript, including abstract, methods, tables, figures, and discussion.

5. 172-173—should be cost per referred child who was reached. It is confusing throughout the manuscript if you are discussing the total number of children who heard the message, or the total TARGET number, which would be those referred. If listing the total number who heard the message, use some space in the discussion to justify this by addressing the benefit of public health awareness beyond the children referred (eg better attitudes towards glasses wear to support those who need them).

Response: We clarified that cost per child who accessed services refers to referred children, while cost per person reached refers to all listeners. In the Discussion, we justify including broader exposure by highlighting the value of mass media campaigns in shaping community norms, citing WHO health promotion guidance.

6: Do not list where to insert the figures. Table 3 labels are not consistently or clearly named

Response: We kept the placeholders to comply to the submission guidelines so that the text could be linked. Figure 2 has been revised to distinguish numbers referred, followed up, and attending care. Table 3 labels have been standardised, e.g. "Cost per referred child accessing care" replaces "Cost per child."

7. Line 239-240 is confusing—how do you know who sought care after only the school broadcast? Isn't this the same children as the combined group, perhaps before the community broadcast? The other portions of the manuscript discuss the 2 groups, but you have divided into 3 groups here.

Response: To clarify, Group 2 does not receive school broadcasts. We conducted three separate difference-in-difference (DiD) analyses, always comparing the same two groups (Group 1 and Group 2. First, we assessed the effect of the school broadcast phase by comparing changes in referral adherence from baseline to Endline 1 between groups. Second, we assessed the effect of the community broadcast phase by comparing changes from Endline 1 to Endline 2, noting that both groups were exposed to community broadcasts but only Group 1 had received prior school exposure. Third, we assessed the combined effect by comparing overall changes from baseline to Endline 2, which reflects school plus community exposure (Group 1) versus community only (Group 2). These analyses did not involve a third study arm; rather, they capture

different stages of exposure within the two-group design. We added this information in Methods (LINE 222 – 232)

8. the abstract is confusing. Don't mention beyond that included in this manuscript. Clarify the intervention.

Response: We revised the Abstract to report only referral adherence outcomes. References to parallel evaluations were removed, though Methods retain a note that they will be reported separately as per the pre-specified plan.

9. Figure 2 numbers don't seem to match the text: 238 of 246 followed up (implies they were referred and received care) would be a 96.7% uptake, and 122 of 128 would be 95.3%

Response: This has now been corrected LINE 293-295.

10. The difference in school size in Group 1 compared to Group 2 may have led to bias as a larger school may be in a larger community, with different healthcare attitudes and behavior. In a separate area, the authors state a higher proportion of rural children in the intervention group, although it is unclear if these are the same schools (large schools in a rural area with large catchment area, vs some large semiurban schools and some rural schools)

Response: We added this as a limitation, noting that larger school sizes and a higher proportion of rural children in the intervention group may have biased comparability.

11. Line 337 wording ("impactful").

Response: Revised to "more impactful."

12. Line 372—analysis of parent and child engagement is not in this manuscript, but rather whether they followed through with seeking care.

Response: We clarified that this manuscript reports only referral adherence outcomes, and engagement analyses will be reported separately.

13. Groups 1 and 2 are not balanced in that the time from screening to the first intervention was much longer in Group 2, which would result in the parent and

child to be less likely to remember the failure and connect it with the content. This may not be adjustable due to the national broadcast, but it's effect should be acknowledged.

Response: We acknowledged this as a limitation. The delay was due to national radio scheduling. We note that a definitive trial will use stratified randomisation and aligned intervention timing to address this.

VERSION 2 - REVIEW

**Reviewer**	**1**
**Name**	**Asare , Afua**
**Affiliation**	**University of Utah Health**
**Date**	**04-Sep-2025**
**COI**	

The manuscript is much improved. I do not have any further concerns

**Reviewer**	**2**
**Name**	**Summers, Allison**
**Affiliation**	**Oregon Health & Science University**
**Date**	**07-Sep-2025**
**COI**	

The authors have thoroughly addressed reviewer questions. The edits are clear and thoughtful. I look forward to this addition to the body of evidence in improving adherence to screening referrals.
